# Tumour vessel remodelling: new opportunities in cancer treatment

**DOI:** 10.1530/VB-19-0032

**Published:** 2020-01-14

**Authors:** Ruth Ganss

**Affiliations:** 1 Vascular Biology and Stromal Targeting, Harry Perkins Institute of Medical Research, The University of Western Australia, Centre for Medical Research, Nedlands, Western Australia, Australia

**Keywords:** cancer and tumours, angiogenesis, vessel normalisation, extracellular matrix, cancer immunotherapy

## Abstract

Tumour growth critically depends on a supportive microenvironment, including the tumour vasculature. Tumour blood vessels are structurally abnormal and functionally anergic which limits drug access and immune responses in solid cancers. Thus, tumour vasculature has been considered an attractive therapeutic target for decades. However, with time, anti-angiogenic therapy has evolved from destruction to structural and functional rehabilitation as understanding of tumour vascular biology became more refined. Vessel remodelling or normalisation strategies which alleviate hypoxia are now coming of age having been shown to have profound effects on the tumour microenvironment. This includes improved tumour perfusion, release from immune suppression and lower metastasis rates. Nevertheless, clinical translation has been slow due to challenges such as the transient nature of current normalisation strategies, limited *in vivo* monitoring and the heterogeneity of primary and/or metastatic tumour environments, calling for more tailored approaches to vascular remodelling. Despite these setbacks, harnessing vascular plasticity provides unique opportunities for anti-cancer combination therapies in particular anti-angiogenic immunotherapy which are yet to reach their full potential.

## Introduction

Cancer and stromal accessory cells co-evolve to foster malignant growth and tumour progression. Among stromal cells, tumour blood vessels have been a major focus in oncology. It has been shown in the early 1970s that the rate of tumour neovascularisation – or angiogenesis – controls tumour growth (1). Subsequently, Folkman’s hypothesis of blocking tumour angiogenesis as a means to starve cancers (2) triggered decades of molecular studies into the pathophysiology of angiogenesis, and most importantly, the development of anti-angiogenesis therapy. In 2004, Bevacizumab (Roche), a humanized antibody against vascular endothelial growth factor (VEGF), became the first anti-angiogenic drug approved in the United States for the treatment of metastatic colon cancer in combination with chemotherapy (3). However, a decade of clinical experience has tempered the initial enthusiasm for anti-angiogenesis therapy. Blood vessel destruction with anti-angiogenic reagents results in transient tumour ‘starvation’ and hypoxia, but in time, adaptive resistance emerges followed by aggressive tumour re-growth (4, 5). Furthermore, in preclinical models, there is clear evidence for enhanced metastatic dissemination with chronic anti-angiogenesis therapy (6).

Coinciding with the idea of killing blood vessels, an alternative concept, namely tumour blood vessel normalisation, first emerged as a strategy to transform the chaotic angiogenic vasculature into a more orderly anatomy which also reduced metastatic dissemination (7). Since then, pioneering studies in the laboratory of Rakesh Jain have delineated molecular processes of tumour vessel remodelling in response to mechanical forces and growth factors within the cancer environment; these studies have deepened our understanding of the tumour vasculature as a barrier to drug delivery (8, 9, 10). In particular, the potential for low-dose anti-VEGF therapy to prune immature tumour vessels and enhance functionality of the remaining vasculature for improved chemo- and radiation therapies was recognized and has shown promising outcomes in preclinical and clinical studies (11). Ganss and colleagues first described the correlation between tumour vessel normalisation and immune cell infiltration (12). In highly angiogenic, non-inflamed cancers, vessel normalisation is necessary and sufficient to enable infiltration by pre-activated immune cells and consequent tumour destruction (13). Subsequently, low-dose anti-VEGF treatment in mouse melanoma was shown to improve adoptive T cell therapy and to re-programme a suppressive innate immune environment (14, 15). More recent evidence suggests that anti-tumour T cells contribute to vessel normalisation in a positive feedback loop where initial T cell infiltration promotes tumour perfusion leading to overall enhanced T cell accumulation and response to checkpoint blockade (16, 17). Thus, at least in animal models, the efficacy of all current anti-tumour therapies, including chemo-, radiation and immunotherapy, is intimately linked to tumour vasculature status, perfusion and oxygenation (18).

## Insights into blood vessel normalisation

**Figure 1:**
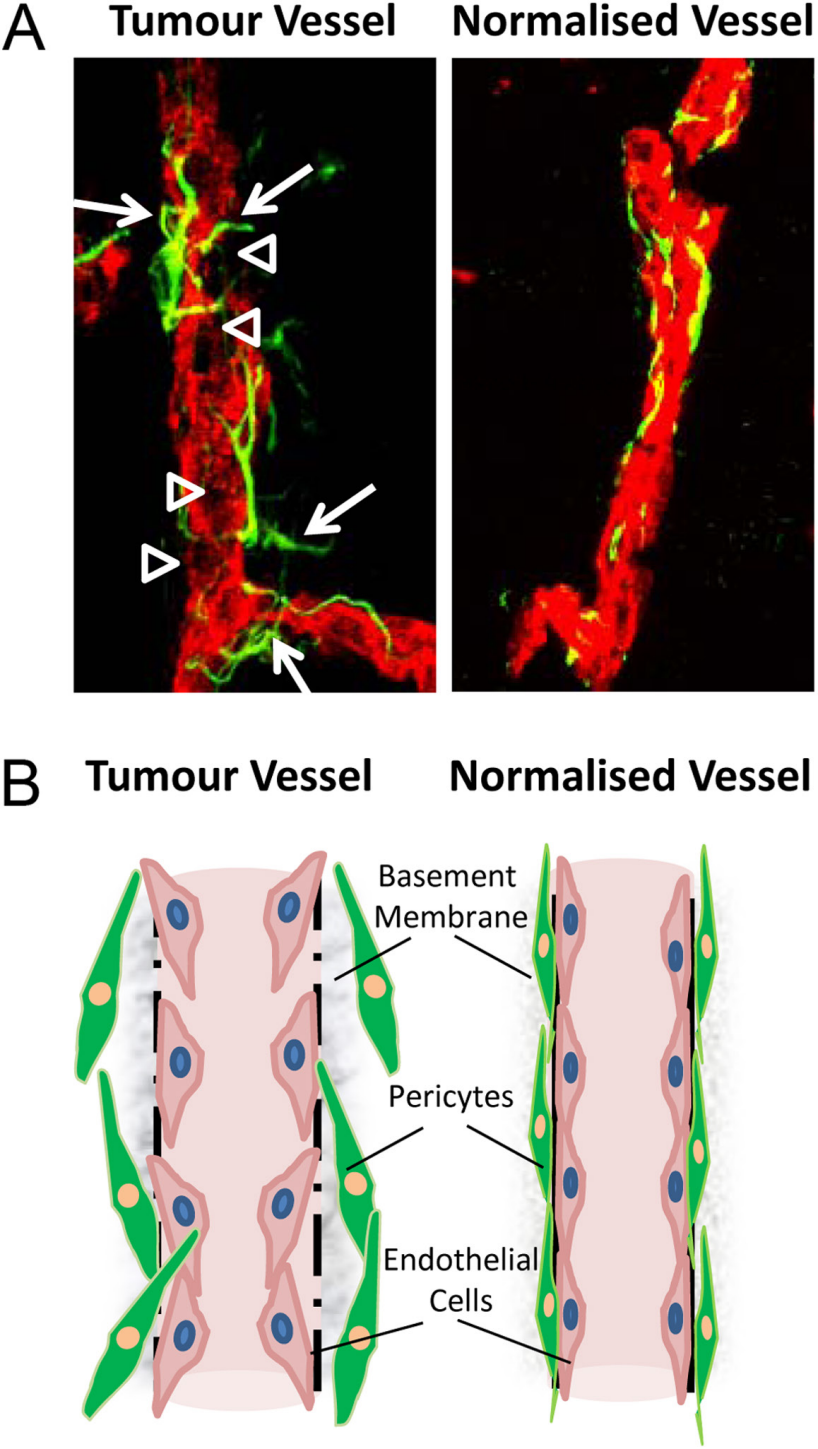
Distinct cellular features of wild type and normalised tumour blood vessels. Tumour blood vessels consist of endothelial cells (inner layer) and, vascular smooth muscle cells or pericytes (outer layer) which are embedded in basement membrane. (A, left) Representative image of a tumour blood vessel featuring disrupted endothelial cell lining (CD31 endothelial marker in red, arrow heads indicated endothelial gaps) and pericytes (desmin pericyte marker in green) protruding into tumour parenchyma as indicated by arrows. (A, right) Representative image of a normalised tumour blood vessel consistent of compact CD31^+^ endothelium and closely aligned desmin^+^ pericytes. Here, normalisation of the entire vascular bed was achieved by changing the maturity of pericytes only. Confocal images, magnification, 60×. (B, left) Schematic representation of a ‘leaky’ tumour blood vessel featuring endothelial cell gaps and irregularly attached pericytes. (B, right) Schematic representation of a normalised tumour blood vessel with closely aligned endothelial cells and pericytes embedded in basement membrane.

Tumours harbour a tortuous network of leaky blood vessels which lack the hierarchical order and patency of their normal counterparts. Tumour blood vessel normalisation restores vascular function thereby increasing tumour perfusion and alleviating hypoxia. This in turn increases the response to therapy, suppresses endothelial-to-mesenchymal transition and reduces metastatic dissemination (19, 20). Blood vessels consist of an inner endothelial cell layer surrounded and supported by mural cells such as pericytes. Endothelial cells and pericytes are normally closely attached and embedded in a mesh of extracellular matrix (ECM) called the basement membrane. In highly leaky cancer vessels, however, pericytes are not well aligned with endothelial cells and indeed migrate away from the compromised vessel wall featuring altered basement membrane thickness and/or composition (21, 22, 23) (Fig. 1). During the vessel normalisation process, disorganized and highly proliferating tumour endothelial cells become more quiescent and form tighter connections between neighbouring cells involving adherens junction molecules such as vascular endothelial (VE)-cadherin (24). In addition, endothelial cells of a normalised vasculature are supported by higher numbers of pericytes or pericytes which are more mature and adhesive (13, 20, 22). While most normalising drugs target the endothelial compartment, therapeutic induction of pericyte quiescence and maturity has similar normalising effects on the entire vascular bed (13, 25). Mechanistically, many factors which regulate cellular differentiation during physiological angiogenesis are also important for tumour vessel normalisation, for instance, angiopoietins (Ang) and their receptors, notch receptors and ligands, and integrins; the role of these molecules in vessel normalisation has been extensively reviewed (10, 11). In a broader context, rendering endothelial cells more quiescent by targeting metabolic or hypoxic response pathways matures the vasculature and increases tumour perfusion (26, 27). Endothelial cells, in particular sprouting vessels, heavily depend on glycolysis for energy production. Reducing endothelial cell glycolysis, for instance, by deleting the glycolytic activator *Pfkfb3* (phosphofructokinase-2/fructose-2,6-biphosphatase 3 enzyme) re-establishes endothelial adhesion and overall vessel maturation (20). Similarly, gene deficiency of *Phd2* (prolyl-hydroxylase) or its upstream regulator *Siah2* (E3 ubiquitin ligase) normalizes vessels by regulating hypoxia-inducible factor (HIF) availability which increases tumour perfusion and pericyte coverage (19, 28). Overall, these functional studies demonstrate that the vessel normalisation process is intimately linked to cell proliferation, differentiation and metabolic function.

## Anti-angiogenic strategies and vascular normalisation

To date, most mechanistic insights into vessel normalisation have been generated following inhibition of VEGF signalling pathways by using moderate-to-low doses of monoclonal antibodies or small-molecule inhibitors targeting tyrosine kinase receptors (29). However, since VEGF is an essential survival factor for endothelial cells, chronic inhibition even at low dose ultimately leads to vessel death or upregulation of other angiogenic factors (15). Induction of more durable normalisation effects therefore necessitates alternative strategies. Indeed, newer drugs which simultaneously block the de-stabilizing Tie-2 receptor ligand Ang-2 and VEGF (CrossMab A2V or Vanucizumab, Roche) or activate Tie-2 whilst blocking Ang-2 using bispecific antibodies potentiate the degree of vessel normalisation in preclinical studies (30, 31, 32) (Table 1). A phase I clinical study of single-agent Vanucizumab in solid cancers (33) and a phase II study in metastatic colorectal cancer comparing Vanucizumab in combination with chemotherapy with Bevacizumab/chemotherapy have been concluded (NCT02141295). Moreover, at a preclinical level, direct targeting of VE-Cadherin by using, for instance, the oligonucleotide-based inhibitor CD5-2 which disrupts the interaction of VE-cadherin with its regulator miR-27a affects multiple junctional proteins and also activates the stabilizing Tie-2-Ang1 pathway, thus re-establishing endothelial barrier function (24). Alternatively or in addition to endothelial cell targeting, forced pericyte maturation by inhibiting PDGF-B signalling using a single-stranded nucleic acid oligonucleotide (aptamer AX102) or local TGFβ stimulation following pericyte-targeted cytokine therapy (LIGHT-VTP, TNFSF14 conjugated to a vascular targeting peptide) effects durable vessel normalisation and improves tumour perfusion in a variety of preclinical models (25, 34). Thus, there is an ever-increasing list of reagents with the capacity to normalise tumour vessels. Which approaches will find their way into the clinic will ultimately depend on delivery efficacy, specificity and durability of normalising effects to maximize the therapeutic window in combination therapies.

**Table 1:** Selected vascular remodelling therapies with synergistic or alternative outcomes to VEGF/VEGFR targeting.

Compound	Specificity	Target/outcome	Tumour type	Preclinical/clinical trial	Reference
CrossMab A2A or Vanucizumab	Bi-specific anti-angiopoetin-2 and anti-VEGF antibody	EC targeting/Vessel normalisation	Breast cancer Melanoma PNET	Preclinical	(31)
			Advanced solid tumours	Phase I	(33)
			Metastatic colorectal cancer	Phase II Combination CT	NCT02141295
			Locally advanced or metastatic solid tumours	Phase I Combination IT	NCT01688206
ABTAA	Angiopoetin-2 binding and Tie2 activating antibody	EC targeting/Vessel normalisation	Glioblastoma Lung cancer Breast cancer PNET	Preclinical	(32)
CD5-2	Oligonucleotide inhibitor interrupts miR-27a-VE-Cadherin interaction; activates Tie2-Ang1	EC targeting/Vessel normalisation	Colon cancer Melanoma PNET	Preclinical	(24)
NGR-TNFα	Peptide-cytokine fusion compound, binds the tumour-specific CD13 splice variant	EC targeting/Vessel normalisation	Melanoma	Preclinical	(55)
			Metastatic melanoma	Phase I Combination IT	(56)
AX102	Oligonucleotide inhibitor for PDGF-B	PC targeting/Vessel normalisation	Lung cancer PNET	Preclinical	(34)
LIGHT-VTP	Peptide-cytokine fusion compound, binds to angiogenic pericytes	PC targeting/Vessel normalisation, HEV and TLS induction	Lung cancer PNET Glioblastoma	Preclinical	(43, 44)
Cilengide combined with Verapamil	αvβ3/αvβ5 integrin binding reagent combined with calcium channel blocker	EC targeting/Increased vessel density and improved blood flow	Lung cancer PDAC	Preclinical	(35)
Losartan	Angiotensin II receptor antagonist	CAF targeting/Reduced ECM production, vascular decompression and improved blood flow	PDAC	Preclinical	(36)
			PDAC	Phase II CNCT/CR	(60)
Vismodegib	Sonic hedgehog signalling inhibitor	CAF targeting/Reduced ECM, improved blood flow	Breast cancer	Preclinical	(38)
PEGPH20 (pegylated hyaluronidase)	Hyaluronic acid	ECM targeting/Reduced ECM, improved blood flow	PDAC	Preclinical	(39)
			Stage IV PDAC	Phase III Combination CT	NCT02715804
TNFα-CSG	Peptide-cytokine fusion compound, binds cancer ECM	ECM targeting/Immune-mediated ECM degradation, vascular decompression and improved blood flow	Breast cancer, PNET	Preclinical	(40)

CAF, cancer-associated fibroblast; EC, endothelial cells; ECM, extracellular matrix; HEV, high endothelial venule; LIGHT, homologous to lymphotoxin, exhibits inducible expression and competes with HSV glycoprotein D for binding to herpesvirus entry mediator, a receptor expressed on T lymphocytes (or TNFSF14); PC, pericytes; PDAC, pancreatic adenocarcinoma; PDGF, platelet-derived growth factor; PNET, pancreatic neuroendocrine tumours; Tie2, tyrosine kinase with immunoglobulin-like and EGF-like domains; TLS, tertiary lymph node structure; TNFα, tumour necrosis factor α; VEGF, vascular endothelial growth factor; VTP, vascular targeting peptide; CT, chemotherapy; IT, immunotherapy; CNCT/CR, combination neoadjuvant chemotherapy/ chemoradiotherapy

## Variations on the theme: tumour vessel remodelling beyond vessel normalisation

**Figure 2:**
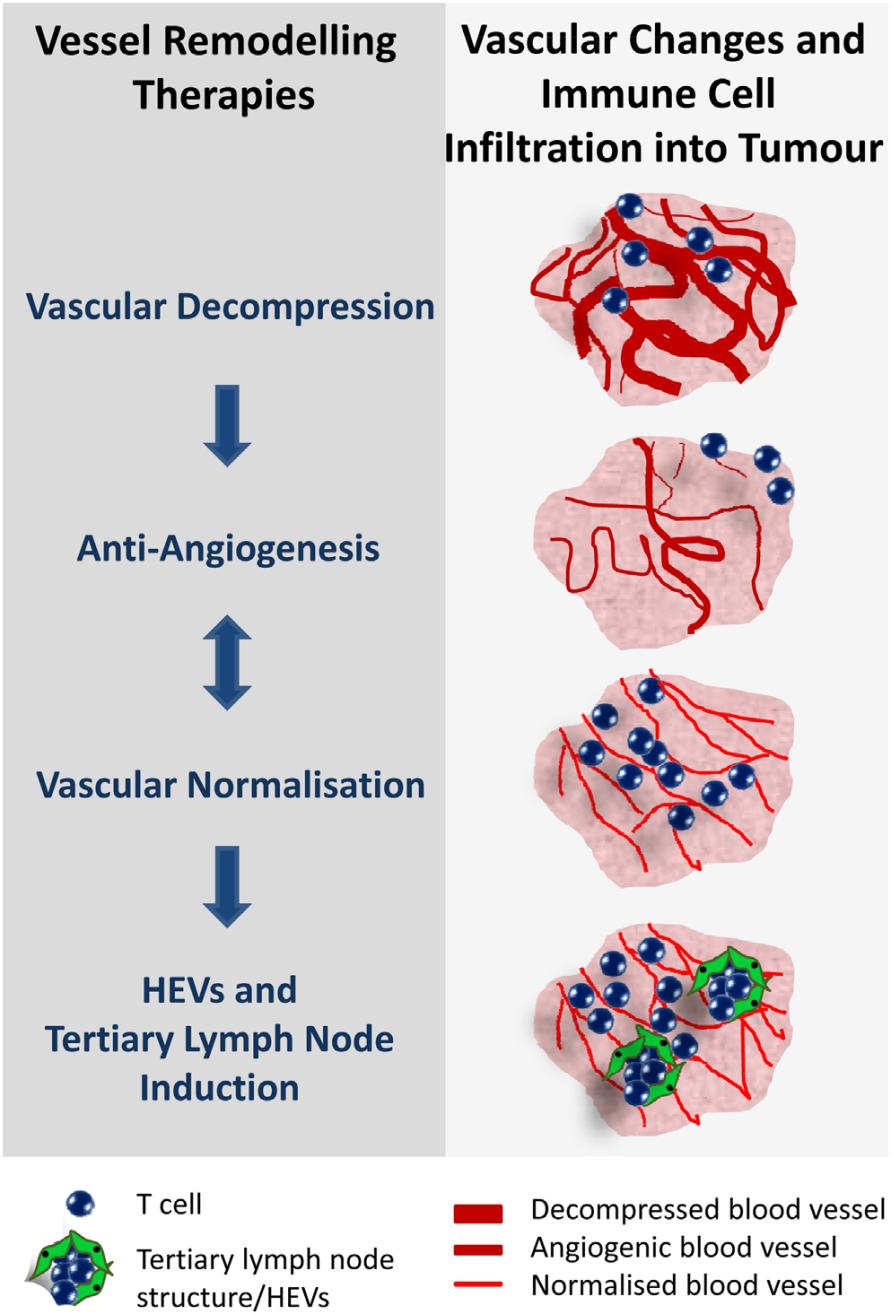
Vessel remodelling strategies to increase tumour perfusion and immune cell penetration. (Left) Therapeutic approaches which aim to destroy or remodel highly angiogenic tumour blood vessels. These approaches are not necessarily mutually exclusive; vessel normalisation and decompression can result in vessel death, and remaining vessels can be normalised during anti-angiogenesis therapy, and induction of high endothelial venules (HEVs) is facilitated on a background of normalised vessels. (Right) Schematic representation of vascular plasticity following therapy and implications for immune cell infiltration. Vascular decompression therapy enlarges blood vessels by alleviating pressure from surrounding extracellular matrix/basement membrane which increases blood flow and potentially immune cell infiltration. Anti-angiogenesis therapy prunes highly proliferative tumour vessels leading to overall blood vessel loss, increase in hypoxia and reduced adaptive immune responses. Vessel normalisation therapy induces a homogeneous vascular network of more quiescent/mature vessels which facilitate infiltration of anti-cancer immune cells. Tertiary lymph node structures, including HEVs, can be therapeutically induced on a background of normalised tumour vessels which increase influx and functionality of adaptive immune cells in the tumour microenvironment.

In addition to vessel normalisation, other vascular remodelling concepts have emerged which are designed to increase vascular function, in particular, in desmoplastic cancers with collapsed blood vessels such as pancreatic adenocarcinoma (PDAC) (Fig. 2). For instance, vascular promotion therapy aims to increase blood vessel density and blood flow while reducing hypoxia. This was achieved in preclinical models of lung and pancreatic cancers by co-administration of low-dose Cilengitide (an αvβ3/αvβ5 integrin-specific RGD-mimetic cyclic peptide) and Verapamil (a generic calcium channel blocker) which increased delivery and responsiveness to chemotherapy (35) (Table 1). Similar in concept, vascular decompression therapy eliminates excessive ECM around blood vessels, increases blood flow and potentiates chemotherapy. For instance, the anti-hypertensive drug Losartan (angiotensin II receptor antagonist) reduces stromal collagen and hyaluronan production in pancreatic adenocarcinoma by inhibiting TGFβ production in cancer-associated fibroblasts (CAFs) (36). Pirfenidone, an anti-fibrotic drug approved for idiopathic pulmonary fibrosis, is similarly effective in reducing stromal TGFβ signalling and increasing perfusion in breast cancer (37). The sonic-hedgehog pathway inhibitor Vismodegib (Roche) improves blood flow and chemotherapy effectiveness by reducing the number of proliferating CAFs and overall tumour collagen and hyaluronan content in breast cancer (38). Direct targeting of hyaluronic acid with pegylated hyaluronidase (PEGPH20, Halozyme Therapeutics) shows improvement of vessel patency in preclinical models (39) and is currently investigated in hyaluronic acid-high, stage IV pancreatic cancer patients in phase III in combination with standard of care chemotherapy (NCT02715804). Specific targeting of tumour ECM using the TNFα-CSG fusion compound attracts immune cells into the tumour microenvironment which secrete a cocktail of proteases to degrade ECM, enlarge tumour vessels and increase perfusion (40).

A different form of tumour vessel remodelling is the induction of high endothelial venules (HEVs), a cell type which is morphologically and functionally distinct from endothelial cells. HEVs are cuboidal in shape and decorated with peripheral node addressin (PNAds) which mediate L-selectin^+^ lymphocyte trafficking in peripheral lymph nodes and at sites of chronic inflammation. HEVs can arise spontaneously in cancer and are associated with a better patient prognosis (41). Importantly, HEVs can also be induced therapeutically, for instance, by the cytokine LIGHT (or TNFS14) and its receptors LTβR/HVEM, a process which is greatly facilitated by a normalised tumour vasculature (42, 43, 44). Since HEVs are entrance portals for lymphocytes, intratumoral HEVs in conjunction with normalized blood vessels in cancer are highly significant for immunotherapy, in particular, for ‘cold’ tumours which lack effector T cell infiltration (45, 46). Overall, stromal changes such as vessel normalisation, activation, trans-differentiation, de-compression or ultimately death demonstrate the plasticity of the vascular bed which can be therapeutically exploited (Fig. 2). While changes in tumour vasculature are not necessarily mutually exclusive and can occur simultaneously or consecutively, a combination of drugs may be required to optimize intratumoral effects in different tumour environments.

## Tumour vessel normalisation and immunotherapy

The advent of checkpoint inhibitors and other immunotherapeutics has changed the oncology landscape profoundly. Impressively, with combined anti-CTLA4 (Ipilimumab, Bristol-Myers-Squibb) and anti-PD1 (Nivolumab, Bristol-Myers-Squibb) treatment, 60% of metastatic melanoma patients now experience a median survival of 2 years rather than 6 months (47). However, checkpoint inhibitors are not universally beneficial. To increase response rates within and across tumour types, an unprecedented number of combination therapies are currently being tested. So far, there is strong preclinical evidence that stromal remodelling agents enhance checkpoint blockade and other immunotherapies (24, 31, 43, 48, 49, 50). This is not overly surprising, since vessel normalisation or decompression reduces hypoxia and enhances T cell trafficking (18, 48); a higher density of intratumoral effector T cells in turn increases the effectiveness of checkpoint blockade (16, 45). In the context of anti-VEGF therapy, however, there are other reasons why vascular remodelling and checkpoint inhibition enhance tumour immunity synergistically. Blocking of pro-angiogenic factors such as VEGF changes the immune suppressive tumour environment by reducing the frequency of alternatively activated macrophages, myeloid suppressor cells and regulatory T cells while enhancing effector T cell function (51). Moreover, anti-angiogenic therapy can also restore the expression of endothelial adhesion molecules, thereby reversing vessel ‘anergy’ and enabling more productive lymphocyte-endothelial interactions (52). Given the profound effects of VEGF inhibition in the tumour microenvironment beyond vascular remodelling, combinations of VEGF-targeting agents and checkpoint inhibitors have rapidly advanced from phase I to III clinical trials with noteworthy early results in renal cell carcinoma and non-small-cell lung cancer (53).

Besides inhibition of VEGF signalling, other diverse strategies have been developed to specifically eliminate physical barriers to effector T cell penetration by targeting vascular and ECM features. For instance, CrossMab A2V (Vanucizumab) normalises angiogenic vessels and also stimulates tumour immunity leading to enhanced anti-PD1 effects (31). Phase I clinical trials combining Vanucizumab with anti-PD-L1 antibodies (Atezolizumab, Roche) are ongoing (NCT01688206). Peptide-mediated cytokine delivery specifically to tumour vasculature, for instance TNFα (RGR-TNFα, NGR-TNFα), normalises and activates endothelial cells, thus increasing adoptive T cell and vaccination therapies (54, 55); first in man studies (phase I, NGR-hTNF, MolMed) have been conducted combining NGR-TNFα and anti-tumour vaccination in metastatic melanoma patients (56). Retrospective analysis of metastatic urothelial cancer patients treated with anti-PD-L1 antibodies (Atezolizumab) demonstrated that TGFβ plays a central role in T cell exclusion and lack of responsiveness (57). Blockade of TGFβ using Galunisertib (TGFβ receptor I inhibitor) in murine colorectal cancer enabled T cell infiltration and responsiveness to PD-L1 therapy (58). Furthermore, therapeutic induction of HEVs triggers formation of distinct lymphocyte clusters or tertiary lymph node structures. These lymph node structures provide a critical microenvironment for generating anti-tumour immune responses and sensitize tumours to checkpoint inhibitor therapy in preclinical models of breast and pancreatic cancers and glioblastoma (43, 49).

Thus, there is a clear correlation between vessel/stromal remodelling and T cell infiltration (18). However, it remains unresolved whether enhanced lymphocyte migration following vessel remodelling requires active receptor-ligand interactions or is regulated by passive mechanisms such as reduced interstitial pressure and hypoxia. It is conceivable though that vascular and ECM remodelling strategies will work in synergy to eliminate physical barriers in the tumour microenvironment and that mechanism-guided combination treatments could greatly improve immunotherapy.

## Clinical challenges

Normalised tumour vessels have been described in many preclinical studies. However, clinical evidence correlating vessel remodelling with better survival outcomes is still sparse. For instance, treatment with VEGF receptor tyrosine kinase inhibitors enhanced survival in those glioblastoma patients who also showed increased tumour perfusion as measured by MRI (59). Neoadjuvant treatment of PDAC consisting of FOLFIRINOX (fluorouracil, leucovorin, oxaliplatin and irinotecan) and losartan followed by chemoradiotherapy achieved a 61% curative resection rate in a phase II trial, possibly linked to improved tumour perfusion (36, 60) (Table 1). Limited patient data reflect the need for further studies of vessel normalisation as an antitumour approach. Challenges include timing and dosing of vessel remodelling agents as well as monitoring of changes in the tumour microenvironment. Moreover, heterogeneity in angiogenic growth factor expression levels and the co-existence of blood vessels with different maturation states within the same tumour lesion impact on therapeutic responses (61). Longitudinal monitoring of tumour vessel status, perfusion, and oxygenation will be required to accurately assess the clinical benefits in combination therapies. Circulating biomarkers, for instance, soluble VEGFR1, Ang2, collagen IV and apelin have been validated in some studies but so far no single predictive marker has been identified, even in the context of anti-VEGF therapy (11, 62). Imaging modalities such as dynamic MRI, blood oxygenation level-dependent (BOLD) MRI or PET are useful technologies to indirectly monitor vascular function and oxygen status but difficult to implement into clinical routine (62). Indeed, current insufficiencies in routine monitoring provide a strong incentive to develop alternative, more robust and durable normalisation strategies to increase the therapeutic window. Moreover, the vast majority of vessel remodelling agents to date are administered systemically. Systemic delivery of VEGF inhibitors for instance can cause off-target effects in healthy tissue, and cessation of anti-VEGF therapy has even been reported to trigger liver metastases (63). Thus, more tailored tumour-targeting strategies may be required which utilize antibodies or peptides to enable deeper and more homogeneous access into tumour parenchyma, as well as simultaneous or sequential targeting of multiple stromal components (64).

## Conclusions

Fifty years after Judah Folkman demonstrated the critical role of tumour angiogenesis, blood vessels remain an attractive target in the tumour microenvironment. The focus, however, has shifted from vessel destruction to remodelling in response to evidence demonstrating that vessel normalisation and tumour oxygenation are intertwined and crucial for combination therapies. To date, clinical insights into anti-angiogenesis/vessel normalisation therapies are still mainly based on VEGF/VEGFR inhibition. Yet, even after a decade in the clinic, the mode of action, selection of responsive patient populations, treatment timeline and mechanisms of drug resistance remain largely unresolved. However, more recently, the immuno-modulatory effects of anti-VEGF therapy have highlighted the intimate relationship between tumour blood vessels and anti-cancer immunity leading to ongoing clinical trials combining VEGF and checkpoint blockade (53). Given the heterogeneity of cancer environments, including highly desmoplastic stroma and lack of immune cell infiltration, new approaches which remove intratumoral barriers and increase T cell trafficking into ‘cold’ tumours are particularly attractive. In the future, vessel and stromal remodelling with more specific and sustained intratumoral effects are likely to become an essential part of combination therapies, in particular, immunotherapies. Monitoring the effects of multiple therapeutic interventions will be crucial for clinical translation.

## Declaration of interest

The author declares that there is no conflict of interest that could be perceived as prejudicing the impartiality of this review.

## Funding

This work was supported by National Health and Medical Research Councilhttp://dx.doi.org/10.13039/501100000925 of Australia (APP1122108, APP1141849) and the Cancer Council Western Australiahttp://dx.doi.org/10.13039/501100001170 (APP1098579). R Ganss is supported by a Cancer Research Institute Clinic and Laboratory Integration Program (CLIP) Grant, Tour de Cure Senior Research Grant and a Woodside Energy Fellowship.
